# Golgi defect as a major contributor to lysosomal dysfunction

**DOI:** 10.3389/fcell.2024.1386149

**Published:** 2024-04-24

**Authors:** Sarah R. Akaaboune, Yanzhuang Wang

**Affiliations:** Department of Molecular, Cellular and Developmental Biology, University of Michigan, Ann Arbor, MI, United States

**Keywords:** HexA, Golgi, GRASP55, GRASP65, lysosome, lysosomal storage diseases, neurodegenerative diseases, secretion

## Abstract

The Golgi apparatus plays a crucial role in lysosome biogenesis and the delivery of lysosomal enzymes, essential for maintaining cellular homeostasis and ensuring cell survival. Deficiencies in Golgi structure and function can profoundly impact lysosomal homeostasis, leading to various lysosomal storage diseases and neurodegenerative disorders. In this review, we highlight the role of the Golgi Reassembly Stacking Proteins (GRASPs) in the formation and function of the Golgi apparatus, emphasizing the current understanding of the association between the Golgi apparatus, lysosomes, and lysosomal storage diseases. Additionally, we discuss how Golgi dysfunction leads to the secretion of lysosomal enzymes. This review aims to serve as a concise resource, offering insights into Golgi structure, function, disease-related defects, and their consequential effects on lysosomal biogenesis and function. By highlighting Golgi defects as an underappreciated contributor to lysosomal dysfunction across various diseases, we aim to enhance comprehension of these intricate cellular processes.

## 1 Introduction

The Golgi apparatus, also known as the Golgi complex, was discovered by the Italian physician scientist Camillo Golgi in the late 19th century (Ludford, 1924; Palade and Claude, 1949). It forms a stack of flattened membranous sacs or cisternae, typically consisting of several cisternae, although the number can vary depending on the cell type and function (Shorter and Warren, 2002; Wang and Seemann, 2011). The Golgi stack comprises the *cis*-Golgi, the region closest to the endoplasmic reticulum (ER) and serves as the entry or receiving side of the Golgi apparatus; the *medial*-Golgi, structures located between the *cis*- and *trans*-Golgi that play a central role in modifying and processing proteins and lipids; and the *trans*-Golgi, the exit side of the Golgi where fully processed molecules are packaged into vesicles for delivery to various cellular destinations (Guo et al., 2014).

The importance of the Golgi in cellular function has been extensively studied (Goldfischer, 1982). The Golgi apparatus is responsible for various functions in eukaryotic cells, including protein and lipid processing, sorting, packaging, trafficking, and secretion (Farquhar and Palade, 1998). One primary function of the Golgi is post-translational modifications of proteins, such as glycosylation, phosphorylation, and proteolytic cleavage (Huang and Wang, 2017). These modifications are essential for their proper structure and physiological function (Zhang and Wang, 2016). It is also well documented that the Golgi apparatus is actively involved in lipid metabolism and synthesis of complex lipids such as glycolipids and sphingolipids (Maccioni et al., 2011; Quinville et al., 2021).

The Golgi also plays an important role in the delivery of lysosomal enzymes, formation of lysosomes, and maintenance of cellular homeostasis (Bajaj et al., 2019). The sorting and packaging of post-translationally modified proteins and lipids involve the formation of various transport vesicles that bud from the *trans*-Golgi network (Guo et al., 2014). These vesicles, containing cargo molecules, are then directly delivered to their intended destinations in various intracellular organelles within the cell, such as endosomes and lysosomes, the plasma membrane, or the extracellular space (Huang and Wang, 2017). It is also well-established that the Golgi apparatus is indispensable for the secretion of essential molecules, including hormones, enzymes, and various proteins, through regulated and/or constitutive secretion mechanisms (Huttner et al., 1995). For instance, in neuronal cells, upon stimulation, vesicles containing proteins transiently stored within the cell fuse with the plasma membrane and release their contents into the extracellular space (Wu et al., 2014; Rudolph et al., 2015). A crucial role of the Golgi apparatus in constitutive secretion, ensuring a continuous supply of essential molecules from the cell to the extracellular environment, has also been documented (Burgess and Kelly, 1987; Bauerfeind and Huttner, 1993). Additionally, the Golgi apparatus is involved in regulating the lipid composition, which is crucial for the integrity, fluidity, and function of cell membranes (Agliarulo and Parashuraman, 2022).

Lysosomes are membrane-bound organelles that contain various hydrolytic enzymes that break down different types of biomolecules. Lysosomes are known for their high dynamics, capable of fusing with membrane vesicles originating from endocytosis, autophagocytosis, and phagocytosis (Bajaj et al., 2019; Amaral et al., 2023). Lysosomes play a crucial role in cellular waste disposal, digestion of foreign materials, recycling of cellular components, and signaling. Lysosome dysfunction can lead to lysosomal storage diseases (LSDs).

LSDs are a group of rare inherited disorders characterized by defects in lysosomal function, leading to the accumulation of undegraded substances within lysosomes throughout the body. Lysosomes are cellular compartments containing enzymes that break down various biomolecules, such as carbohydrates, lipids, and proteins (Samie and Xu, 2014). In LSDs, genetic mutations result in the deficiency or malfunction of specific lysosomal enzymes, preventing the proper breakdown of these substances (Ren and Wang, 2020). Consequently, undegraded materials accumulate within lysosomes, causing cellular dysfunction and impairing the functioning of organs and tissues (Bajaj et al., 2019). The diverse array of LSDs manifests in a spectrum of symptoms, including neurological impairment, skeletal abnormalities, organ dysfunction, and other systemic complications. These disorders often present challenges for diagnosis and management due to their rarity, complexity, and variability in clinical manifestations (Sun, 2018; Bajaj et al., 2019). Though individually rare, as a group, LSDs underscore the critical role of lysosomes in maintaining cellular homeostasis and the severe consequences that result from their dysfunction (Samie and Xu, 2014).

While the role of genetic mutations in causing LSDs has been extensively investigated, the impact of Golgi defects observed in diseases on lysosomal biogenesis and function has been somewhat overlooked in the research field. This review aims to consolidate current knowledge on the mechanism of Golgi structure formation, function, and defects in diseases. We then explore the connection between the Golgi and lysosomes under physiological and pathological conditions. By drawing attention to Golgi defects as an underappreciated contributor to lysosomal dysfunction across various diseases, we seek to enhance comprehension of these intricate cellular processes, particularly by investigating Golgi defects in human diseases associated with LSDs not linked to genetic mutations.

## 2 GRASP proteins and their impact on Golgi structure, function, and disease-related defects

The Golgi apparatus has been extensively studied in recent decades (Farquhar and Palade, 1998). With proteomics, engineered genetics, and biochemical approaches, several essential structural proteins associated with this cellular organelle were identified (Slusarewicz et al., 1994; Nakamura et al., 1995; Wu et al., 2000). The stacking of the Golgi apparatus is a complex and dynamic process involving a series of proteins, including Golgins, Golgi Reassembly and Stacking Proteins (GRASPs), Golgi matrix proteins, microtubules, and motor proteins (Xiang and Wang, 2011; Zhang and Wang, 2015b). All these proteins are essential for the formation and preservation of the structural integrity and shape of the Golgi apparatus within eukaryotic cells (Glick and Nakano, 2009; Wang and Seemann, 2011).

In mammalian and other eukaryotic cells, the Golgi apparatus consists of flattened cisternae (membrane-enclosed sacs) that originate from vesicular clusters budding off the ER (Jensen and Schekman, 2011), and Golgi stacks are interconnected by tubular membrane structures to form a ribbon (Klumperman, 2011). Golgi ribbon formation requires the integrity of a microtubule network (Wei and Seemann, 2010). Without these connections, the Golgi would exist as individual stacks dispersed in the cytoplasm. In plant cells, however, Golgi stacks are linked by actin filaments rather than microtubules (Driouich et al., 2012). In this section, we will focus on the role of GRASPs in the structure and function of the Golgi under physiological and pathological conditions. The role of Golgins, Golgi matrix proteins, and microtube/motor proteins in the preservation of Golgi stacks was reviewed elsewhere (Short et al., 2005; Lowe, 2011; Xiang and Wang, 2011).

### 2.1 GRASP proteins and Golgi structure formation

**FIGURE 1:**
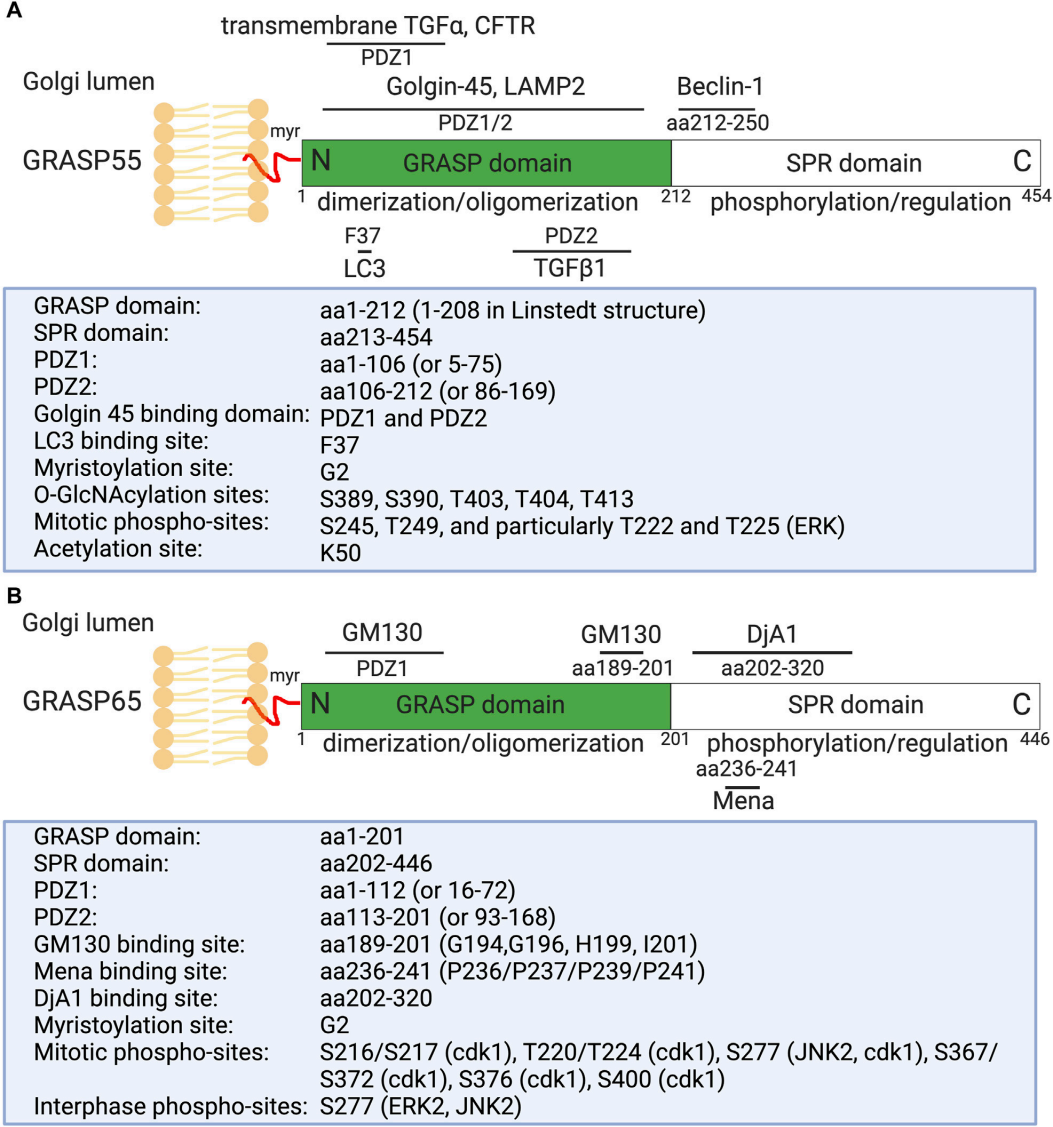
Domain structure, modification, and binding sites on GRASP55 **(A)** and GRASP65 **(B)**. Rat GRASP55 and GRASP65 sequences are used for illustration. Both GRASPs exhibit a similar structural organization: a conserved N-terminal GRASP domain comprising two PDZ domains (PDZ1 and PDZ2), and a C-terminal Serine/Proline-Rich (SPR) domain featuring multiple phosphorylation sites, which play crucial roles in GRASP regulation throughout the cell cycle. GRASP55 is also acetylated on K50; deacetylation is required for postmitotic Golgi reassembly. GRASP65 and GRASP55 are both peripheral membrane proteins anchored to the Golgi membranes via N-terminal myristoylation and interaction with their respective membrane-bound partner proteins (GM130 and Golgin-45). Mena and DjA1 are GRASP65-binding proteins identified to enhance Golgi ribbon linking and stacking, respectively. GRASP55 undergoes regulation by O-GlcNAcylation in response to glucose levels and interacts with LC3 and LAMP2 to facilitate autophagy induction during glucose starvation [modified from (Ahat et al., 2019a; Li et al., 2019a)].

**FIGURE 2:**
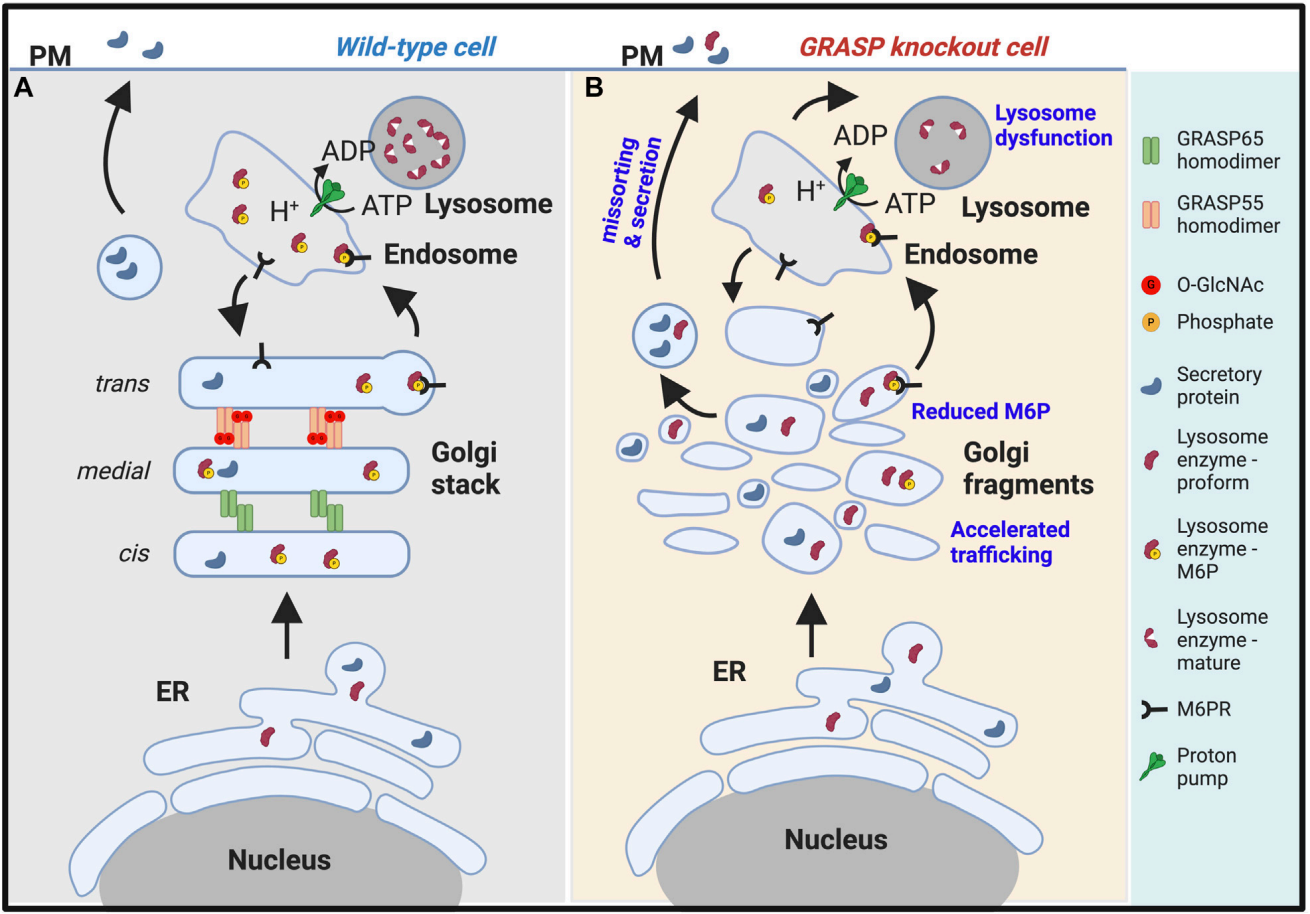
Golgi fragmentation causes lysosomal dysfunction by missorting and secretion of lysosomal enzymes. **(A)**. In wildtype cells, GRASP55 and GRASP65 maintain the stacked Golgi structure and ensure correct protein sorting. While both lysosomal enzymes and secretory proteins are synthesized in the endoplasmic reticulum (ER), they are processed differently in the Golgi. Lysosomal enzymes modified by mannose-6-phosphate (M6P), which serves as a sorting signal, are recognized by the M6P receptor (M6PR) and delivered to lysosomes via endosomes. Secretory proteins are transported through the Golgi and to the plasma membrane (PM) for secretion (Xiang et al., 2013; Zhang and Wang, 2015b). **(B)**. In GRASP knockout cells, particularly GRASP55 knockout cells (Ahat et al., 2022a), the Golgi stacked structure is disrupted, and mannose-6-phosphorylation of lysosomal enzymes becomes less efficient, leading to missorting and secretion of lysosomal enzymes. Under this condition, only the proform of lysosome enzymes are secreted. Consequently, lysosomes become dysfunctional due to the lack of lysosomal hydrolases (Zhang and Wang, 2016; 2020a).

GRASPs, initially identified as Golgi stacking factors (Barr et al., 1997; Shorter et al., 1999), have been shown to play crucial roles in the formation and maintenance of Golgi stacks and the overall function of the Golgi apparatus (Zhang and Wang, 2015b; Rabouille and Linstedt, 2016). They have the capability to link adjacent cisternae by forming *trans*-oligomers, effectively tethering one cisterna to another in an orderly fashion (Wang et al., 2003; Xiang and Wang, 2010; Jarvela and Linstedt, 2014). Two main GRASP proteins have been identified: GRASP55 (molecular weight 55 kDa) and GRASP65 (molecular weight 65 kDa) (Figure 1) (Barr et al., 1997; Shorter et al., 1999). These proteins display distinct localizations within the Golgi stack (Shorter et al., 1999). GRASP65 predominantly localizes to the *cis*-Golgi, while GRASP55 localizes to the *medial*- and *trans*-cisternae (Figure 2A) (Shorter et al., 1999).

Structural analysis of GRASP55 and GRASP65 revealed that these proteins share similar conserved two PDZ domains (PDZ1 and PDZ2) at the N-terminus (Figure 1). These conserved PDZ domains facilitate the ability of GRASP to form dimers and *trans*-oligomers between stacks and to enable interactions with various Golgi proteins, such as GM130 (associated with GRASP65) and Golgin-45 (associated with GRASP55) (Hu et al., 2015; Zhao et al., 2017), as well as enzymes and cytoskeletal components, which are critical for the organization and function of the Golgi apparatus (Ahat et al., 2019a; Li et al., 2019a). GRASP55 and GRASP65, however, differ in the serine proline-rich (SPR) domain at the C-terminus, which contains several phosphorylation sites that play an important role in Golgi dynamics during the cell cycle or under pathological conditions (Wang et al., 2005; Vielemeyer et al., 2009; Tang et al., 2012; Joshi et al., 2014).

Recently, several new GRASP-binding proteins have been identified. For instance, GRASP65 has been shown to interact with the actin elongation factor Mena (mammalian-enabled homolog) and the Hsc70 co-chaperone DjA1 (DnaJ homolog subfamily A member 1) (Figure 1) (Tang et al., 2016; Li et al., 2019b). Mena-GRASP65 interaction promotes actin polymerization and GRASP65 oligomerization in Golgi structure formation (Tang et al., 2016). DjA1 was initially identified as a GRASP65-interacting protein through affinity purification and mass spectrometry. Depletion of DjA1 in cells led to Golgi fragmentation, characterized by short and improperly aligned cisternae, as well as delayed Golgi reassembly following nocodazole washout. DjA1 is known as a co-chaperone of Heat shock cognate 71 kDa protein (Hsc70) that also indirectly interacts with GRASP65 through DjA1. Initially, it was speculated that DjA1 facilitated GRASP65 folding. However, subsequent experiments ruled out this notion, demonstrating that DjA1’s role in Golgi structure formation is independent of its co-chaperone activity or Hsc70. Despite being recognized primarily as an Hsc70 co-chaperone, DjA1 directly interacts with GRASP65 to facilitate its oligomerization and promote Golgi stack formation in an Hsc70-independent manner, revealing a novel function of this protein (Li et al., 2019b).

On the other hand, GRASP55 can interact with Microtubule-associated protein one light chain 3 (LC3) on autophagosomes and LAMP2 on lysosomes to facilitate autophagosome-lysosome fusion (Zhang et al., 2018; Zhang and Wang, 2018b; a). GRASP55 also interacts with Beclin-1 (BECN1) to facilitate the UVRAG phosphatidylinositol 3-kinase (PI3K) complex formation and membrane association (Zhang et al., 2019b). Moreover, GRASP55 is required for unconventional secretion of cystic fibrosis transmembrane conductance regulator (CFTR) and transforming growth factor beta 1 (TGFβ1) (Gee et al., 2011; Nuchel et al., 2018). These interactions highlight the diverse roles of GRASP proteins in cellular processes beyond Golgi structure maintenance, involving functions in actin dynamics, autophagy, and unconventional protein secretion (Zhang and Wang, 2020b).

Several studies have documented that the tethering of Golgi cisternae in an ordered manner by GRASP proteins is essential for the proper function of the Golgi. This organized tethering and stacking process is likely required for the precise and sequential posttranslational modifications of proteins and lipids as they move between cisternae, facilitating efficient processing and sorting (Zhang and Wang, 2015b; Huang and Wang, 2017). Experiments of inhibition and depletion of GRASP proteins have played a crucial role in elucidating the functions of GRASPs in Golgi stacking and trafficking processes. For example, inhibiting GRASPs’ function through microinjecting GRASP antibodies, knocking down GRASPs expression levels with siRNA, or depleting GRASPs from cells using the CRISPR/Cas9 approach led to significant alterations in Golgi stacks, including Golgi fragmentation (Wang et al., 2003; Tang et al., 2010b; Bekier et al., 2017). It is noteworthy that a cell-based study concluded that acute GRASP depletion did not affect Golgi stacking (Zhang and Seemann, 2021). However, the average number of cisternae per stack in this study was about 4, significantly lower than what is typically seen in various cell lines. Based on the literature, the number of cisternae within a stack varies between 4 and 11 in mammalian cells (Rambourg and Clermont, 1997). In our electron microscopy (EM) studies of the Golgi, we consistently observe that the typical number of cisternae per Golgi stack is approximately five to six in HeLa cells (Tang et al., 2010b; Xiang and Wang, 2010; Tang et al., 2016; Bekier et al., 2017; Li et al., 2019b; Ireland et al., 2020), CHO cells, and primary cultured hippocampal neurons (Joshi et al., 2014). Importantly, it appears that this study only counted properly aligned stacked membranes, which may have impacted the conclusion. In another study, mice deficient in either GRASP55 or GRASP65 exhibited limited defects in Golgi structure and function (Veenendaal et al., 2014; Chiritoiu et al., 2019). One potential concern regarding the GRASP65 knockout mouse strain is the presence of mRNA encoding exon one to three, which encodes a 115 aa N-terminal fragment of GRASP65. If this fragment is translated, it could potentially be sufficient for Golgi stacking (Tang et al., 2010b). Alternatively, the organized Golgi structure may be attributed to the redundancy of the other GRASP protein, which could compensate for the knockout effect of one GRASP. Indeed, it has been shown that, in cells where one GRASP is depleted, the level of the remaining GRASP protein may increase to offset the knockout effect (Bekier et al., 2017; Ahat et al., 2019a). In cells, the expression of phosphorylation-deficient mutants of GRASP55 and GRASP65 has been shown to increase the number of cisternae per stack in interphase cells and to inhibit the disassembly of Golgi stacks in mitosis (Wang et al., 2005; Tang et al., 2010b; Xiang and Wang, 2010). Overexpression of either GRASP55 or GRASP65 alone in HEK293 cells transfected with a plasmid containing the deleted phenylalanine-508 (ΔF508) mutation in the cystic fibrosis transmembrane conductance regulator (CFTR) could induce the surface expression of ΔF508-CFTR and rescue chloride channel activity. In ΔF508-CFTR transgenic mice, overexpression of GRASP55 led to a significant improvement in the survival of the mice (Gee et al., 2011). These findings suggest that GRASP proteins play dual roles in Golgi structure formation and unconventional trafficking of CFTR.

### 2.2 Cell cycle regulation of Golgi membrane dynamics via GRASP proteins

The Golgi apparatus is a highly dynamic organelle capable of adapting its structure to various cellular signals (Bankaitis et al., 2012; Tang and Wang, 2013). During mammalian cell division, Golgi stacks undergo disassembly and subsequent reassembly after division (Wang and Seemann, 2011; Tang and Wang, 2013). The mechanism by which GRASP proteins control the unstacking and stacking of Golgi cisternae has been extensively investigated. Converging data indicate that phosphorylation and dephosphorylation events of GRASP proteins play crucial roles in the disassembly and reassembly of Golgi stacks (Tang et al., 2010a). GRASP proteins undergo phosphorylation during cell division, leading to their de-oligomerization and subsequent disassembly of Golgi stacks. After cell division, GRASP proteins are dephosphorylated, facilitating the formation of GRASP65 oligomers and the subsequent reassembly of Golgi cisternae (Wang et al., 2003; Wang et al., 2005; Xiang and Wang, 2010).

These findings were further substantiated by experiments demonstrating that phosphorylation of GRASP by recombinant Cyclin dependent kinase 1 (Cdk1)/cyclin B1 and Polo-like kinase (Plk1) in mitosis induces the disassembly of GRASP oligomers, resulting in unstacking of Golgi cisternae (Figure 1) (Wang et al., 2005; Tang et al., 2010a). In cells overexpressing mutant GRASP proteins lacking the phosphorylation sites, the mitotic Golgi disassembly of stacks is inhibited, as GRASP proteins cannot undergo de-oligomerization (Wang et al., 2005; Tang et al., 2010b). Similarly, in *Drosophila* cells, knockdown of dGRASP (the sole GRASP protein in *Drosophila*) using RNAi induces the disassembly of Golgi stacks (Kondylis et al., 2005). Phosphorylation of GRASP65 by JNK2 has been demonstrated to control Golgi fragmentation at the G2/M transition (Cervigni et al., 2015). Furthermore, GRASP65 has been identified as a regulator of spindle dynamics, playing an essential role in cell division (Sutterlin et al., 2005).

The role of GRASP55 in Golgi stacking is also regulated by Sirtuin 2 (SIRT2) (Zhang et al., 2019a), a NAD-dependent sirtuin deacetylase that involves various cellular processes, such as microtubule and chromatin dynamics, gene expression, cell cycle progression, and nuclear envelope reassembly (North et al., 2003; Pandithage et al., 2008; Janke, 2014; Kaufmann et al., 2016). Depletion of SIRT2 in cells induces Golgi fragmentation and impairs Golgi reassembly at the end of mitosis due to acetylation of GRASP55 (Zhang et al., 2019a). During mitosis, SIRT2 interacts with highly acetylated GRASP55, regulating its acetylation levels. When expressed in GRASP55 and GRASP65 double-knockout cells, both wild-type (WT) and acetylation-deficient mutant of GRASP55, but not an acetylation mimetic mutant, successfully restored the Golgi structure and facilitated post-mitotic Golgi reassembly. Notably, the acetylation-deficient mutant of GRASP55 showed a higher self-interaction efficiency, which is essential for Golgi structure formation. These findings highlight the regulatory role of SIRT2 in Golgi structure through the modulation of GRASP55 acetylation at the end of mitosis (Zhang et al., 2019a).

In addition to GRASP proteins, other Golgi proteins are regulated by other mechanisms during the cell cycle. For example, the SNARE protein syntaxin-5 (Huang et al., 2016) is regulated by ubiquitination mediated by the HECT domain and ankyrin repeat-containing E3 ubiquitin protein ligase 1 (HACE1) (Tang et al., 2011) and the deubiquitinase valosin-containing protein (VCP) complex-interacting protein 135 kDa (VCIP135) (Wang et al., 2004; Zhang et al., 2014; Zhang and Wang, 2015a). The monoubiquitination and deubiquitination cycle regulates p97/VCP-mediated Golgi membrane fusion (Rabouille et al., 1995; Uchiyama et al., 2002); disruption of this cycle impairs post-mitotic Golgi membrane fusion (Zhang et al., 2015; Jiang et al., 2022). GM130 is known to be phosphorylated by Cdk1 at the onset of mitosis (Lowe et al., 1998), which regulates spindle assembly (Kodani and Sutterlin, 2008; Wei et al., 2015). These different modifications of different proteins coordinate with each other to regulate Golgi membrane dynamics during the cell cycle (Wang and Seemann, 2011; Tang and Wang, 2013).

### 2.3 Golgi structure formation controls protein trafficking and processing within the Golgi

Upon the arrival of proteins from the ER into the Golgi, a diverse array of post-translational modifications occurs, including the addition or removal of sugar residues in carbohydrate chains (glycosylation), phosphorylation, sulfation, as well as lipid metabolism and the synthesis of complex lipids such as glycolipids and sphingolipids (Huang and Wang, 2017; Li et al., 2019a; Kweon et al., 2024). In addition to these modifications, the Golgi serves as the focal point for sorting and directing proteins and lipids to their intended destinations within the cell, such as the plasma membrane, lysosomes, or various intracellular organelles, where they can carry out their functions (Bankaitis et al., 2012; Pothukuchi et al., 2021).

As described earlier, GRASP proteins (GRASP55 and GRASP65) are indispensable for Golgi stack formation and are also involved in the trafficking and sorting of proteins across the Golgi (D'Angelo et al., 2009). Depletion of GRASP proteins has been associated with several deficiencies in cargo sorting and trafficking within the Golgi apparatus (Xiang et al., 2013). The intra-Golgi trafficking speed, the accessibility of coat proteins to Golgi membranes, and accurate glycosylation and sorting have all been impacted (Wang et al., 2008; Zhang and Wang, 2015b; 2016). GRASP proteins have been implicated in the regulation of cargo trafficking through direct interaction. Certain proteins, such as TGFα, CD83, CD8α, and Frizzled4, possess a C-terminal valine residue that interacts with the PDZ domains of GRASP proteins (D'Angelo et al., 2009). Additionally, recent findings have demonstrated that some lipid droplet-associated proteins, including adipose triglyceride lipase (ATGL) and monoglyceride lipase (MGL), utilize a Golgi- and GRASP55-dependent pathway to reach lipid droplets. Interestingly, this process requires a direct interaction between GRASP55 and ATGL, despite the absence of a C-terminal valine in these proteins (Kim et al., 2020). Furthermore, GRASP55 can directly bind Golgi enzymes such as glucosylceramide synthase and lactosylceramide synthase, facilitating proper compartmentalization within the Golgi (Pothukuchi et al., 2021). Consequently, GRASP proteins may influence protein trafficking by directly interacting with cargo molecules or by modulating Golgi stacking and vesicle budding.

While the intuitive assumption might be that proper Golgi stacking should increase the trafficking of proteins and Golgi unstacking would decrease the rate of protein trafficking, experiments have shown the opposite (Wang et al., 2008; Xiang et al., 2013). Instead, Golgi unstacking accelerates protein trafficking. For example, the rate of COPI vesicle formation from Golgi membranes was significantly increased as vesicles formed more efficiently from unstacked cisternae (Figure 2B). This process may enhance protein transport through the Golgi apparatus to the cell surface (Wang and Seemann, 2011). Consistent with this idea, studies have shown that the depletion of GRASPs accelerates the trafficking of several marker proteins, including CD8, vesicular stomatitis virus G-protein, cathepsin D, and integrins (Wang et al., 2008; Xiang et al., 2013; Lee et al., 2014; Bekier et al., 2017).

Disruption of the Golgi structure via GRASP depletion also significantly alters post-translational modifications of proteins, including a decrease in glycan abundance, glycan complexity, and glycoprotein composition at the plasma membrane (Xiang et al., 2013; Bekier et al., 2017). It causes missorting of lysosomal enzymes, such as cathepsin D, to the extracellular space (Figure 2B) (Xiang et al., 2013; Zhang and Wang, 2015b). Disruption of the Golgi structure also impairs glycosaminoglycan synthesis, sulfation, and secretion (Ahat et al., 2022b). Subsequently, Golgi structural defects alter higher cellular activities such as cell attachment, migration, and growth (Ahat et al., 2019b). During mitosis, the phosphorylation of α-mannosidase I (ManIA1), the first glycosylation enzyme cargo proteins encounter upon arrival at the Golgi, impairs its interaction with Mgat1, another Golgi glycosylation enzyme, reducing its enzymatic activity. Golgi fragmentation during mitosis disrupts the organized structure of the Golgi apparatus, halting trafficking and leaving the enzymes and substrates within the same membrane compartments for an extended period until trafficking resumes upon mitotic exit. These events collectively contribute to prolonging the interaction between cargo proteins and glycosylation enzymes during mitosis. Mitotic phosphorylation of MAN1A1, the first enzyme that cargo molecules encounter upon arriving at the Golgi, reduces its enzymatic activity. This mechanism serves to prevent over-modification of cargo proteins by Golgi enzymes during cell division (Huang et al., 2022). All these observations collectively suggest that cisternae stacking is fundamentally important for the Golgi function (Zhang and Wang, 2016; 2020a).

### 2.4 The role of the Golgi in lipid metabolism

The Golgi apparatus plays a pivotal role in lipid metabolism, serving as a central hub for synthesizing, modifying, and sorting lipids within the cell (Bankaitis et al., 2012). One of its primary functions is the processing of lipids, including the synthesis of complex sphingolipids and glycolipids. Sphingolipids, a diverse class of lipids, are crucial components of cellular membranes and are involved in various cellular processes, including signal transduction and the formation of membrane microdomains (Simons and Ikonen, 1997). Additionally, glycolipids synthesized in the Golgi contribute to the structural integrity of cell membranes and participate in cell signaling events (Campadelli et al., 1993).

Disruptions in Golgi function can have profound implications for lipid homeostasis and cellular health (Campadelli et al., 1993). Alterations in the Golgi apparatus may lead to dysregulation in the levels of specific lipid species, impacting cellular processes. Studies have shown that Golgi dysfunction can result in changes in the levels of key lipids. Indeed, disruption of the Golgi by knocking out GRASP55 and GRASP65 reduces globotriaosylceramide (Gb3) expression but increases monosialotetrahexosylganglioside (GM1) level (Bekier et al., 2017), highlighting the intricate connections between Golgi function and lipid metabolism. A recent study revealed that GRASP55 selectively binds and compartmentalizes essential glycosphingolipid biosynthetic enzymes. This specific compartmentalization within the Golgi ensures precise biosynthetic reactions and regulates the cellular glycosphingolipid profile (Pothukuchi et al., 2021).

The delicate balance between GM1 and Gb3 is particularly crucial in Golgi stacks, given their distinct roles in cellular processes (Celi et al., 2022). Although both GM1 and Gb3 are crucial for myelination, GM1 has also been shown to promote β-amyloid peptide (Aβ) aggregation and toxicity (Sipione et al., 2020). It is noteworthy that elevated Gb3 level correlates with the development of gastric, colon, and breast cancers, and Gb3 is also implicated in the mechanisms of Epithelial-to-Mesenchymal Transition (EMT) (Zhuo et al., 2018). Additionally, Gb3 has been linked to the susceptibility to chemotherapeutic agents, as evidenced by its increased level at the cell surface in cisplatin-resistant cells (Tyler et al., 2015). These findings further underscore Golgi’s involvement in cancer cell adaptation. Further exploration of Golgi’s role and its defects in lipid regulation holds promise for understanding the underlying mechanisms of lipid-related disorders. Such exploration may unveil potential therapeutic targets for conditions linked to aberrant lipid metabolism.

### 2.5 Golgi defects are observed in human diseases

Golgi structural and functional defects have been observed in various diseases, including Alzheimer’s (Aridor and Balch, 1999; Joshi et al., 2014; Evin, 2015; Joshi et al., 2015; Joshi and Wang, 2015), Huntington’s (Hilditch-Maguire et al., 2000; Ahat et al., 2022a), Parkinson’s (Mizuno et al., 2001), amyotrophic lateral sclerosis (ALS) (Mourelatos et al., 1996; Gonatas et al., 1998; Fujita and Okamoto, 2005), autoimmune diseases (Fritzler et al., 1984; Bizzaro et al., 1999), cancer (Yoshimura et al., 1996; Roberts et al., 1998; Dennis et al., 1999; Diaz-Corrales et al., 2004; Ono and Hakomori, 2004; Krishnan et al., 2005), viral infections (Ng et al., 2003; Cortese et al., 2020; Zhang et al., 2022), congenital disorders of glycosylation (Zeevaert et al., 2008; Lubbehusen et al., 2010), and Wiskott-Aldrich syndrome (Durand and Seta, 2000; Freeze and Ng, 2011). Despite increasing awareness of these associations, the causal relationship between Golgi defects and disease pathogenesis remains largely unexplored. Here, we present a few examples of Golgi defects in human diseases that have been investigated.

#### 2.5.1 Golgi defects in Alzheimer’s diseases

Alzheimer’s disease (AD) is characterized by the formation of two types of protein aggregates in the brain: neurofibrillary tangles (NFTs) in neurons formed by hyperphosphorylated tau and extracellular β-amyloid plaques formed by the accumulation of secreted Aβ (Suh and Checler, 2002; Small and Gandy, 2006). Aβ, a proteolytic product of the amyloid precursor protein (APP), undergoes cleavage by α-, β-, and γ-secretases during the later steps of intracellular transport (Small and Gandy, 2006; Haass et al., 2012). Trafficking and maturation of APP and its processing enzymes require proper functioning of the Golgi apparatus (Thinakaran et al., 1996; Dries and Yu, 2008; Choy et al., 2012; Prabhu et al., 2012). APP and all three secretases are synthesized in the ER and transferred to the Golgi, where they are modified by glycosylation, phosphorylation, and proteolysis (Barlowe et al., 1994; Brandizzi and Barlowe, 2013). Importantly, glycosylation (Tomita et al., 1998) and phosphorylation (Vieira et al., 2009; Barbagallo et al., 2010) in the Golgi affect APP trafficking and processing. The Golgi plays a pivotal role in the assembly of the γ-secretase complex, which includes the catalytic subunit presenilin 1 (PS1) and associated proteins. The complex is formed into active enzymes following PS1 processing and nicastrin glycosylation in the Golgi (Yang et al., 2002; Takasugi et al., 2003).

**FIGURE 3:**
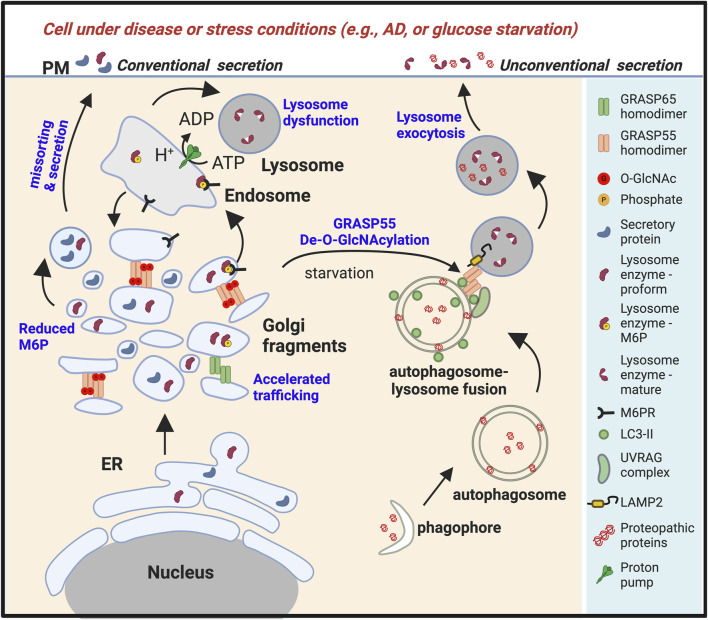
Golgi fragmentation in stress or disease conditions leads to lysosomal dysfunction and unconventional secretion of lysosomal contents. Under stress conditions such as energy and nutrient deprivation (Zhang et al., 2018; Zhang et al., 2019b), or in diseases such as Alzheimer’s disease (AD) or other types of neurodegeneration (Ahat et al., 2022a), or upon viral infection (Zhang et al., 2022), the Golgi undergoes fragmentation, resulting in misssorting of lysosomal enzymes and reduced lysosomal function. Glucose starvation also reduces O-GlcNAcylation of GRASP55. De-O-GlcNAcylated GRASP55 is then targeted to the autophagosome-lysosome interface via interactions with LC3 and LAMP2, facilitating autophagosome-lysosome fusion (Zhang et al., 2018). Stress also induces lysosome exocytosis, leading to the secretion of lysosome contents (Ahat et al., 2022a). Under these conditions, both the proform and the mature form of lysosomal enzymes, together with aggregative cytoplasmic proteins, are secreted.

Significantly, the Golgi architecture is abnormally fragmented in neurons from AD human brain and AD mouse models (Dal Canto, 1996; Stieber et al., 1996; Gonatas et al., 1998; Joazeiro and Weissman, 2000; Huse et al., 2002; Baloyannis, 2014; Joshi et al., 2014; Ayala and Colanzi, 2017; Santos et al., 2017). Numerous pathologies in AD may be related to defects in the Golgi and the secretory pathway, including increased production of the toxic Aβ peptide, abnormal protein glycosylation, and impaired lysosomal/autophagosomal degradation (Figure 3). Investigation into Golgi structure defects in AD reveals that Aβ oligomer accumulation causes Golgi fragmentation by activating Cdk5 and phosphorylating GRASP65. Expression of APPswe and PS1∆E9, or treatment of primary neurons with oligomeric Aβ peptides, leads to Golgi fragmentation. Interestingly, inhibition of β- and γ-secretases, but not α-secretase in AD cells, reduces Golgi fragmentation (Joshi et al., 2014). Rescuing Golgi structure by inhibiting Cdk5 or expressing non-phosphorylatable GRASP mutants reduces Aβ secretion by elevating non-amyloidogenic α-cleavage of APP. These findings highlight Golgi defects as a critical mechanism for Aβ toxicity and demonstrate that rescuing Golgi structure reduces Aβ production by shifting APP cleavage towards the non-amyloidogenic pathway (Joshi et al., 2014; Evin, 2015; Joshi et al., 2015; Joshi and Wang, 2015).

#### 2.5.2 Golgi fragmentation in cancer

The role of the Golgi in cancer cells is a subject of ongoing debate, with the structure and function of this organelle being influenced by cancer hallmarks. The Golgi’s involvement in cancer extends beyond a mere consequence of transformation, actively contributing to malignancy through aberrant glycosylation, enhanced survival, proliferation, and increased migration (Bisel et al., 2008). Abnormal Golgi morphology is a common feature in cancer, ranging from constitutively disassembled units to fragmented or indistinguishable structures (Maldonado et al., 1966; Kellokumpu et al., 2002; Petrosyan, 2015). Recent studies suggest that alteration of Golgi dynamics may contribute to cancer malignancy (Zhang, 2021).

Golgi dispersal is a prevalent feature in various cancer types, including colon, breast, gastric, and prostate cancers (Egea et al., 1993; Sewell et al., 2006; Nolfi et al., 2020). Cancer cell lines often display fragmented Golgi, and tissue sections from cancer patients exhibit distinct Golgi structures compared to non-cancerous cells (Kellokumpu et al., 2002). Golgi disorganization has been linked to altered protein glycosylation, sorting, and functions, all crucial for cell survival, proliferation, and migration-major hallmarks of cancer (Ahat et al., 2019b; Bui et al., 2021; Zhang, 2021).

Alterations in Golgi structure in cancer align with the progression of the disease. Golgi dispersal is linked to mitotic phosphorylation of Golgi structural proteins and kinase activation induced by proinflammatory cytokines, cellular stresses, and growth factors. Aberrant activation of kinases, such as PKCα, Src, ERK8, and Pak1, has been observed in various cancers, contributing to Golgi fragmentation (Ching et al., 2007; Chia et al., 2014; Ireland et al., 2020). The role of Golgi in mitotic Golgi disassembly and its link to cancer cell changes in cell cycle progression are essential areas of investigation.

Elevated expression of Golgi matrix proteins, including GRASP55 and GM130, has been associated with poor prognosis in some cancers, suggesting their importance in tumor cells (Bui et al., 2021; Bucurica et al., 2023). Golgi compaction in cancers has been linked to EMT, a spectrum of hybrid or partial states (Tan et al., 2017a; Tan et al., 2017b). Golgi’s involvement in enhanced extracellular matrix (ECM) secretion during tumorigenesis, regulated by Golgin-45/myosin IIA-containing protein complex and tumor suppressor genes like p53, underscores its central role in cancer biology (Tan et al., 2021).

The Golgi stacking proteins GRASP65 and GRASP55 have also implicated in cell cycle control. Phosphorylation of GRASP65 by Cdk1 and Plk1, along with its role in Golgi ribbon formation, suggests a link to cell cycle progression (Lin et al., 2000; Wang et al., 2003). GRASP55 phosphorylation by ERK2 (Jesch et al., 2001; Xiang and Wang, 2010), a major mitotic kinase, further supports the connection between Golgi structure and cell cycle control. In addition, both GRASP65 and GM130 have been shown to regulate mitotic spindle assembly (Sutterlin et al., 2005; Kodani and Sutterlin, 2008; Wei et al., 2015). In summary, the Golgi’s involvement in cancer is multifaceted, impacting cellular processes crucial for malignancy. Understanding the dynamic regulation of Golgi structure and its molecular interactions in cancer cells provides valuable insights into potential therapeutic targets for cancer treatment.

#### 2.5.3 Golgi fragmentation in SARS-CoV-2 infection

The Golgi apparatus emerges as a key player in the intricate interaction between host cells and the severe acute respiratory syndrome coronavirus 2 (SARS-CoV-2), the cause of the COVID-19 pandemic. The virus employs host cell’s angiotensin-converting enzyme 2 (ACE2) receptor for entry, where the spike (S) protein undergoes activation by transmembrane serine protease 2 (TMPRSS2) (V'Kovski et al., 2021). Following this interaction, the virus fuses with host cell membranes at the cell surface or within endosomes, depending on TMPRSS2 activity. The viral RNA is released into the host cell cytosol, initiating a cascade of events. SARS-CoV-2 engages the host cell’s ER to form double-membrane vesicles (DMVs) that support viral genomic RNA replication (Angelini et al., 2013; Oudshoorn et al., 2017). The Golgi plays a center role in the later stages of the viral infection cycle, serving as the assembly site for three essential structural proteins - spike, envelope (E), and membrane (M). Intriguingly, the SARS-CoV-2 spike protein undergoes glycosylation in the Golgi, influencing its stability, interaction with ACE2, and susceptibility to vaccines (Sanda et al., 2021; Tian et al., 2021). Compared with other SARS-related coronaviruses, the SARS-CoV-2 spike protein possesses a unique furin cleavage site (Chan and Zhan, 2022), which is cleaved in the late Golgi to facilitate membrane fusion and viral entry (Shang et al., 2020).

Preliminary findings underscore the profound impact of SARS-CoV-2 infection on the Golgi, leading to Golgi fragmentation (Zhang et al., 2022). Disrupting Golgi function with small molecules, such as brefeldin A and monensin, significantly reduces viral infection (Zhang et al., 2022). This suggests a critical role of the Golgi in the SARS-CoV-2 lifecycle. Furthermore, the infection results in decreased expression of GRASP55 (Zhang et al., 2022). Intriguingly, expression of various SARS-CoV-2 viral proteins, including spike, ORF3a, M, and E, induces Golgi fragmentation, providing insights into the molecular mechanisms of viral-induced Golgi structural changes (Zhang et al., 2022). As described above, GRASP55 is a known key player in Golgi cisternae stacking (Xiang and Wang, 2010), its reduced expression disrupts the Golgi structure and accelerates protein trafficking (Wang et al., 2008; Xiang et al., 2013). These results indicate that SARS-CoV-2 modulates Golgi structure and function by decreasing GRASP55 expression to facilitate viral trafficking and secretion (Zhang et al., 2022). This finding is significant as it reveals a non-genetic factor affecting viral production and infectivity.

One profound impact of SARS-CoV-2 infection is that it can cause an AD-like neuropathological phenotype and clinical “brain fog” (Fernandez-Castaneda et al., 2022; Zazhytska et al., 2022). SARS-CoV-2 infection and AD are similar in that both diseases induce Golgi fragmentation (Joshi et al., 2014; Zhang et al., 2022). The convergence of SARS-CoV-2 infection and AD pathology at the Golgi raises questions about shared molecular pathways (Wang and Gandy, 2022). Both diseases involve type I membrane proteins, such as the SARS-CoV-2 spike protein and APP, which undergo similar modifications in the Golgi and are impacted by Golgi fragmentation. It is possible that SARS-CoV-2 infection-triggered Golgi fragmentation may potentially accelerate APP trafficking and processing, activating an AD-like program. The observed “brain fog” associated with SARS-CoV-2 infection, reflecting cognitive impairment, adds another layer of complexity. Golgi fragmentation induced by the virus may enhance cytokine secretion, contributing to cognitive symptoms. Additionally, SARS-CoV-2-induced defects in myelination, possibly related to Golgi fragmentation, might explain cognitive impairment, given the Golgi’s role in lipid synthesis (Fernandez-Castaneda et al., 2022). Furthermore, the Golgi’s involvement extends beyond its structure, as Golgi fragmentation may impact distal compartments of the secretory pathway, including autophagosomes and lysosomes (Zhang and Wang, 2020b), as discussed below. Understanding these molecular and cellular dysfunctions is crucial for comprehending the broader implications of Golgi fragmentation in SARS-CoV-2 infection and AD. Exploring the therapeutic potential of GRASPs and Golgi-targeted interventions may provide valuable insights into addressing currently untreatable human diseases (Wang and Gandy, 2022).

## 3 Lysosomes and their roles in cellular homeostasis

### 3.1 Lysosomal functions

A defining feature of lysosomes is their ability to maintain an acidic pH of around 4.5, a critical condition for the optimal activity of hydrolytic enzymes (Amaral et al., 2023). These organelles contain numerous hydrolases that can break down various biomolecules, including proteins, lipids, carbohydrates, nucleic acids, misfolded or damaged proteins and organelles, and cellular debris. This enzymatic breakdown prevents the accumulation of toxic aggregates and upholds proper protein quality control (Chen et al., 2011). The resulting processed materials, such as amino acids and sugars, can either be recycled for reuse or eliminated, ensuring the cell remains devoid of accumulated waste materials that could disrupt cellular function, thereby maintaining cellular homeostasis (Luzio et al., 2014; Bajaj et al., 2019).

Additionally, studies indicate that lysosomes can participate in cellular signaling pathways. They also release certain enzymes and molecules into the cytoplasm or extracellular space, affecting various cellular processes such as autophagy, cell growth, and apoptosis (Bonam et al., 2019). Furthermore, lysosomes contain potent enzymes that regulate lipid metabolism by breaking down complex lipids into their constituent components. This process allows the cell to utilize these molecules for energy or rebuild the membrane (Yim and Mizushima, 2020; Yang and Wang, 2021). In summary, lysosomal function is central to the cell’s waste disposal, protein quality control, lipid metabolism, signaling, and protection against pathogens (Bajaj et al., 2019; Yang and Wang, 2021).

### 3.2 Lysosomal storage diseases

Lysosomal storage diseases (LSDs) constitute a rare group of inherited genetic diseases characterized by impaired lysosomal function (Sun, 2018). While most LSDs follow an autosomal recessive inheritance pattern, some, such as Hunter disease, Fabry disease, and Danon disease, are X-linked inheritance patterns (Rajkumar and Dumpa, 2023). Mutations in genes encoding lysosomal enzymes are the primary cause of LSDs. LSDs encompass over 70 different genetic disorders. Each condition arises due to a deficiency in a specific lysosomal protein or activity (Futerman and van Meer, 2004; Sheth et al., 2023). LSDs are typically progressive and can affect multiple organ systems, including the central nervous system (Rajkumar and Dumpa, 2023). It is important to note that not all mutations result in defective enzymes.

LSDs are classified based on the specific enzyme deficiency or the accumulated material (Sun, 2018; Rajkumar and Dumpa, 2023). There are seven classes of LSDs. 1) Sphingolipidoses, caused by the accumulation of phospholipid materials. This class includes GM2 gangliosidosis (such as Tay-Sachs disease and Sandhoff disease), Niemann-Pick diseases (A, B, and C types), Gaucher disease, Fabry disease, Metachromatic leukodystrophy, Krabbe disease, and multiple sulfatase deficiency. 2) Oligosaccharidoses result from deficiencies in enzymes involved in oligosaccharide breakdown. This class includes alpha-mannosidosis, Schindler disease, Aspartylglucosaminuria, and Fucosidosis. 3) Mucopolysaccharidoses due to the buildup of mucopolysaccharides. Examples include Hurler syndrome, Hunter syndrome, Sanfilippo syndrome, Morquio syndrome, Maroteaux-Lamy syndrome, and Sly syndrome. 4) Neuronal ceroid lipofuscinoses that are characterized by the accumulation of lipopigments in neuronal tissues. 5) Galactosialidosis, which results from defects in enzyme protection proteins. This includes disorders like infantile sialic acid storage disease, Salla disease, and Sialuria. 6) Mucolipidoses, resulting from membrane transport defects, leading to targeting errors. Subtypes include Sialidosis I and II (Mucolipidosis I), I-cell disease (Mucolipidosis II), Pseudo-Hurler-Polydystrophy (Mucolipidosis III), and Mucolipidosis IV. 7) Miscellaneous LSDs, including conditions like Lysosomal Acid Lipase Deficiency (accumulation of cholesterol esters), Pompe disease (glycogen storage disease type II), Danon disease (glycogen), and Cystinosis (cystine) (Schulze and Sandhoff, 2011; Ferreira and Gahl, 2017; Platt et al., 2018).

Studies of fibroblast cells derived from patients with LSDs have played a crucial role in providing information about lysosomal enzyme deficiencies and the classification of LSD types (Filocamo and Morrone, 2011; Venkatarangan et al., 2023). Various forms of enzyme deficiencies have been identified. While many of these disorders are due to enzyme deficiencies, increasing cases have been attributed to issues such as failure to segregate into lysosomes, instability, rapid inactivation, or lack of function due to the absence of activator proteins (Parenti, 2009). Patients sharing the same enzyme deficiency may also possess allelic mutations causing the same defect via diverse mechanisms. This phenomenon is well-documented in Tay-Sachs disease (Barritt et al., 2017; Ramani and Parayil Sankaran, 2023), metachromatic leukodystrophy (Gieselmann et al., 1991; Lamichhane and Rocha Cabrero, 2023), and Pompe disease (Arad et al., 2005; Vellodi, 2005; van der Ploeg and Reuser, 2008).

LSDs comprise a diverse range of clinical phenotypes, and the severity of symptoms can vary significantly even within the same disorder type. For example, mucolipidosis II (ML-II) and mucolipidosis III (ML-III) are LSDs that exhibit different levels of deficiency in GlcNAc-1-phosphotransferase activity. Extremely low or undetectable enzyme levels characterize ML-II, whereas ML-III shows some residual phosphorylating activity (Khan and Tomatsu, 2020). Certain patients exhibit increased secretion of multiple lysosomal enzymes within ML-III, while others display highly elevated plasma lysosomal enzymes (Oussoren et al., 2018; Khan and Tomatsu, 2020). Due to the deficiency of phosphotransferase activity in these patients, the phosphomannosyl recognition marker on glycosylated enzymes cannot be synthesized, preventing their proper targeting to lysosomes. Consequently, some enzymes are secreted into the extracellular milieu instead of being directed to lysosomes (Oussoren et al., 2018; Khan and Tomatsu, 2020). Interestingly, not all cells deficient in phosphotransferase activity lead to the mistargeting of lysosomal enzymes. In certain cell types like hepatocytes, Kupffer cells, and leukocytes, there are nearly regular levels of lysosomal enzymes, suggesting an alternative targeting mechanism distinct from the M6P pathway (Sly, 2000). Moreover, in ML-III fibroblasts, it has been observed that the affinity of phosphotransferase for lysosomal enzymes is markedly reduced, indicating that these patients may possess normal levels of phosphotransferase but with impaired recognition function (Oussoren et al., 2018; Khan and Tomatsu, 2020).

In the case of Pompe disease (glycogenosis type II), it has been observed that the acid alpha-glucosidase enzyme is produced in normal quantities as a precursor form (110-kD). However, the mature 76-kD enzyme is either completely absent or highly inefficient (Reuser et al., 1995). In all reported cases of Pompe disease, it has been observed that glycosylation of the mutant precursors remains unaffected. This implies that either the glycosylated precursor fails to move from the ER to the Golgi apparatus, where phosphorylation typically occurs, or that the mutation leads to a loss of recognition by the phosphotransferase within the Golgi (Meena and Raben, 2020; Taverna et al., 2020).

Similarly, in Tay-Sachs disease, a lysosomal storage diseases caused by the loss of function of the enzyme β-hexosaminidase A (HEXA), it has been reported that the alpha-chain precursor of hexosaminidase is glycosylated normally but not phosphorylated in the Golgi. This indicates that the mutation may prevent the transport of the precursor out of the ER (Barritt et al., 2017; Ramani and Parayil Sankaran, 2023). In the case of Fabry disease, a lysosomal storage diseases it is characterized by the deposition of lysosomal glycosphingolipids due to the absence or deficient activity of lysosomal exoglycohydrolase α-galactosidase A (α-D-galactoside galactohydrolase). This leads to the gradual buildup of Gb3 (or GL-3) and related glycosphingolipids within lysosomes across different cell types (Lenders and Brand, 2021; Cortes-Saladelafont et al., 2023).

## 4 Interplay between the Golgi and lysosomes under physiological and pathological conditions

Due to the dynamic nature of the Golgi and lysosomes within the same endomembrane system, the interplay between the Golgi apparatus and lysosomes is essential for maintaining cellular homeostasis and ensuring proper cellular function. Under physiological conditions, these organelles work in concert to regulate various cellular processes, including protein trafficking, sorting, and degradation. However, disruptions in the Golgi-lysosome axis can occur under pathological conditions, leading to dysregulation of cellular processes and the onset of various diseases. In this section, we explore the dynamic relationship between the Golgi and lysosomes, both in normal cellular physiology and in the context of disease pathology. We discuss how Golgi structure and function alterations impact lysosomal biogenesis, enzyme trafficking, and lysosome-related diseases. Additionally, we discuss the role of Golgi dysfunction in the pathogenesis of neurodegenerative disorders and LSDs, highlighting the interconnectedness of these organelles in health and disease.

### 4.1 The importance of Golgi-dependent mannose 6-phosphate (M6P) pathway in lysosomal enzymes targeting and biogenesis

Lysosomal enzymes, as well as secretory and plasma membrane proteins, undergo cotranslational glycosylation with preformed N-linked oligosaccharides in the ER, typically consisting of three glucose, nine mannose, and two N-acetyl glucosamine residues, attached to specific asparagine residues (Helenius and Aebi, 2001). Following signal sequence cleavage, the protein mixture is transported to the Golgi apparatus, undergoing additional posttranslational modifications (Stanley, 2011). Subsequently, these proteins are sorted for targeting to their appropriate destinations, such as lysosomes, secretory granules, and the plasma membrane (Zhang and Wang, 2016).

In the Golgi apparatus, the path of lysosomal enzymes diverges from that of other glycoproteins. Golgi enzymes process most of the N-linked oligosaccharides on lysosomal enzymes, leading to the acquisition of phosphomannosyl residues (Staudt et al., 2016). This mannose 6-phosphate (M6P) recognition marker is generated through a two-step reaction catalyzed by two Golgi enzymes. First, UDP-GlcNAc: lysosomal enzyme N-acetylglucosamine-1-phosphate transferase (GNPTAB) transfers N-acetylglucosamine-l-phosphate to selected mannose residues on lysosomal enzymes, forming a phosphodiester intermediate (Bajaj et al., 2019). Subsequently, alpha-N-acetylglucosaminidase removes the N-acetylglucosamine, exposing the Man-6-P monoester signal (Bajaj et al., 2019; Amaral et al., 2023). It is worth noting that phosphotransferase can selectively phosphorylate lysosomal enzymes as opposed to non-lysosomal glycoproteins that harbor similar oligosaccharides. This discrimination is achieved by recognizing a protein domain common to nearly all lysosomal enzymes (Luzio et al., 2014; Amaral et al., 2023).

After the generation of phosphomannosyl residues, lysosomal enzymes bind with high affinity to mannose 6-phosphate receptors (M6PR) in the Golgi, facilitating their segregation from proteins destined for secretion (Figure 2A) (Coutinho et al., 2012). Subsequently, the ligand-receptor complex exits the Golgi via clathrin-coated vesicles and enters endosomes (Guo et al., 2014). Within this compartment, ligand dissociation is prompted by acidification, allowing the receptor to recycle back to the Golgi for subsequent binding with another ligand molecule (Bajaj et al., 2019; Amaral et al., 2023). A recent study employing a genome-wide CRISPR/Cas9 knockout screen revealed that the transmembrane protein 251 (TMEM251) plays a pivotal role in modulating M6P modification. TMEM251, localized in the Golgi apparatus, is indispensable for the cleavage and function of GNPTAB, the enzyme responsible for catalyzing M6P modification. Deletion of TMEM251 results in the mistargeting of most lysosomal enzymes due to the absence of M6P modification, leading to the accumulation of undigested materials (Zhang et al., 2022; Pechincha et al., 2022; Richards et al., 2022). Furthermore, in zebrafish models, TMEM251 deletion induces severe developmental anomalies reminiscent of Mucolipidosis Type II (Zhang et al., 2022).

While the phosphorylation of lysosomal enzymes with M6P is crucial for targeting numerous enzymes to lysosomes, some mammalian cell types do not phosphorylate lysosomal enzymes. Nevertheless, these enzymes are correctly targeted to lysosomes, suggesting the existence of other unknown mechanisms (Cuozzo and Sahagian, 1994). For example, the glucocerebrosidase lysosomal enzyme lacks phosphorylation on its oligosaccharide, yet it remains firmly bound to the membrane while targeting the lysosome (Aerts et al., 1988; Braulke et al., 2023).

The M6PR has been purified, and its localization has been investigated through EM and other immunological approaches (Hoflack and Kornfeld, 1985; Pohlmann et al., 1989). However, the precise localization of the M6PR within the Golgi apparatus was controversial. Brown and Farquhar, using an immunoperoxidase technique coupled with EM, found that receptors are restricted to the *cis* side of the Golgi stacks, supported by the presence of the two enzymes involved in generating phosphomannosyl residues within the Golgi stack (Brown and Farquhar, 1984). In contrast, Geuze and others, utilizing ultra-thin cryosections and double-label immune-EM with colloidal gold particles of varying sizes, discovered that the M6PR is localized throughout both *cis* and *trans*-Golgi cisternae (Geuze et al., 1984). This distribution is supported by the co-localization of the lysosomal enzyme cathepsin D with the M6PR in all Golgi cisternae. This suggests that M6PR/cathepsin D complexes move to lysosomes through the entire Golgi complex (Geuze et al., 1984).

Lysosomal enzymes are synthesized as inactive pro-proteins, and their activation is a tightly regulated process crucial for their proper function within lysosomes. The nascent lysosomal enzymes typically contain a prodomain, which acts as a molecular switch to keep them inactive during synthesis and transport. The prodomain prevents premature enzyme activity and protects other cellular components from potential damage. The prodomain must be selectively removed to render these inactive enzymes fully functional. This maturation process occurs within the acidic environment of the lysosome, where specific proteases, often other lysosomal enzymes, cleave the prodomain, activating the enzyme (Figure 2A). For example, Cathepsin D originates as a 53-kDa polypeptide precursor synthesized in the ER, and it is then transported through the Golgi to reach the lysosome. Once within the lysosome, the precursor is converted into a 47-kDa intermediate form, which is further processed into the 31-kDa mature form. (Gieselmann et al., 1985; Zaidi et al., 2008). The precise cleavage of the prodomain is vital for controlling the timing and location of enzyme activation, ensuring that these powerful hydrolytic enzymes become active only within the lysosomal compartment. Dysregulation of this maturation process can lead to lysosomal dysfunction and contribute to the pathogenesis of LSDs. This information is crucial, considering that a proprotein is typically secreted directly from the Golgi, implying that a Golgi defect could potentially impair its sorting and trafficking (Figure 2). Conversely, the secretion of a mature lysosome enzyme usually indicates enhanced lysosome exocytosis (Figure 3).

### 4.2 Golgi structural defects affect lysosome biogenesis and function

As discussed above, the Golgi plays a critical role in lysosome biogenesis and function by controlling essential processes such as M6P phosphorylation, sorting, and delivery of enzymes to lysosomes. Furthermore, the proper functioning of the Golgi is vital for the glycosylation, processing, assembly, and trafficking of lysosomal membrane proteins, including Lamp1, Lamp2, lysosomal ion channels, and transporters. Disruption of Golgi structure formation, induced by GRASP depletion, results in the misplacement of lysosomal enzymes, such as cathepsin D, into the extracellular milieu (Xiang et al., 2013; Zhang and Wang, 2015b). It is noteworthy that only the proform of cathepsin D is secreted upon GRASP depletion, confirming that it is due to a sorting defect at the Golgi (Figure 2), not lysosome exocytosis (Figure 3).

The secretome analysis of WT and GRASP55 knockout (55KO) cells provides valuable insights into the impact of GRASP55 on protein secretion, particularly those associated with lysosomes (Ahat et al., 2019b). Through Tandem Mass Tag (TMT) labeling and liquid-chromatography mass spectrometry (LC-MS), 1,696 proteins were identified in the conditioned media, and their secretion patterns were compared between 55KO and WT cells. Further exploration of the 445 proteins significantly affected by 55KO highlighted distinct secretion patterns based on the presence or absence of ER signal sequences. Notably, proteins without ER signal sequences were less secreted; whereas proteins with ER signal sequences, including lysosomal enzymes, exhibited increased secretion in 55KO cells (Ahat et al., 2019b). This suggests a critical role of GRASP55 in the secretion of lysosomal components, impacting lysosomal biogenesis, consistent with the observation that GRASP depletion disrupts the Golgi structure and accelerates protein trafficking and secretion (Xiang et al., 2013). Unlike secretory proteins that follow the conventional ER-Golgi-plasma membrane pathway, cytosolic proteins lacking ER signal sequences utilize an unconventional autophagosome-autolysosome-plasma membrane pathway for transport. GRASP55 has been shown to facilitate autophagosome-lysosome fusion (Zhang and Wang, 2018b; Zhang et al., 2018), thereby promoting the secretion of cytoplasmic proteins. Additionally, stress-induced Golgi fragmentation may impair the sorting of lysosomal enzymes. Consequently, lysosomal dysfunction cause by lysosome enzyme missorting could diminish the degradation of cytoplasmic proteins in autolysosomes, potentially leading to their increased secretion (Xiang et al., 2013; Zhang and Wang, 2020b; Ahat et al., 2022a).

Gene Ontology (GO) term analysis highlights the importance of lysosomal enzymes and structural proteins in the GRASP55-dependent secretome. This finding aligns with the concept that Golgi unstacking due to GRASP depletion leads to missorting and elevated secretion of lysosomal enzymes (Figure 2B) (Xiang et al., 2013). The identified enzymes in this context include Arylsulfatase B (ARSB), Cathepsin A (CTSA), Cathepsin B (CTSB), Cathepsin C (CTSC), Cathepsin D (CTSD), Cathepsin F (CTSF), Cathepsin L (CTSL), Cathepsin S (CTSS), Cathepsin V (CTSV), Cathepsin Z (CTSZ), HEXA, Hexosaminidase B (HEXB), and N-acetylgalactosamine-6-sulfatase (GALNS), among numerous other lysosomal enzymes (Table 1) (Ahat et al., 2019b).

**TABLE 1:** Lysosomal enzymes with enhanced secretion due to GRASP55 knockout. Associated diseases sourced from GeneCards. Secretion effects represented as fold changes [log2FC(55KO/WT media)] and *p*-values based on prior secretome analysis (Ahat et al., 2022a).

Gene Symbol	Full Name	Function	Associated disease	log2FC (55KO/WT media)	*p*-value
AGA	Aspartylglucosaminidase	Lysosomal breakdown of glycoproteins	Aspartylglucosaminuria and Lysosomal Storage Disease	1.0473	5.11E-03
ARSA	Arylsulfatase A	Hydrolyzes cerebroside sulfate to cerebroside and sulfate	Metachromatic leucodystrophy (MLD)	1.0813	3.03E-03
ARSB	Arylsulfatase B	Hydrolyzes sulfate groups of N-Acetyl-D-galactosamine, chondriotin sulfate, and dermatan sulfate	Mucopolysaccharidosis, Type Vi and Mucopolysaccharidosis Type 6, Slowly Progressing	1.2963	5.35E-04
ARSK	Arylsulfatase Family Member K	hydrolyze sulfate esters from sulfated steroids, carbohydrates, proteoglycans, and glycolipids	Mucopolysaccharidosis, Type X and Mucopolysaccharidosis-Plus Syndrome	0.8600	9.08E-04
ASAH1	N-Acylsphingosine Amidohydrolase 1	Catalyzes the degradation of ceramide into sphingosine and free fatty acid	Farber Lipogranulomatosis and Spinal Muscular Atrophy With Progressive Myoclonic Epilepsy	0.8230	5.15E-03
CPQ	Carboxypeptidase Q	Catalyzes the hydrolysis of dipeptides with unsubstituted terminals into amino acids	Episodic Ataxia Type 4	0.6963	6.55E-02
CTSB	Cathepsin B	Lysosomal cysteine protease with both endopeptidase and exopeptidase activity	Keratolytic Winter Erythema and Annular Erythema	0.9350	4.92E-03
CTSC	Cathepsin C	Lysosomal cysteine proteinase, activates many serine proteinases in cells of the immune system, degrades glucagon	Papillon-Lefevre Syndrome and Haim-Munk Syndrome	1.0063	2.77E-03
CTSF	Cathepsin F	A papain family cysteine protease that participates in intracellular degradation and turnover of proteins	Ceroid Lipofuscinosis, Neuronal, 13 and Neuronal Ceroid Lipofuscinosis	0.9113	3.48E-03
CTSL	Cathepsin L	Lysosomal cysteine proteinase, degrades collagen and elastin, as well as S1 subunit of the SARS-CoV-2 spike protein	Middle East Respiratory Syndrome and COVID-19	0.7843	1.52E-02
CTSV	Cathepsin V	A lysosomal cysteine proteinase that may play an important role in corneal physiology	Endochondral ossification with skeletal dysplasias	1.2883	2.67E-04
CTSZ	Cathepsin Z	A lysosomal cysteine proteinase that exhibits both carboxy-monopeptidase and carboxy-dipeptidase activities; expressed in cancer cells	Cercarial Dermatitis and Rosacea	1.0747	3.46E-03
DNASE2	Deoxyribonuclease 2, Lysosomal	Hydrolyzes DNA under acidic conditions	Autoinflammatory-Pancytopenia Syndrome and Transient Neonatal Thrombocytopenia	1.2037	1.69E-03
FUCA1	Alpha-L-Fucosidase 1	Lysosomal enzyme involved in the degradation of fucose-containing glycoproteins and glycolipids	Fucosidosis and Nervous System Disease	0.8787	1.44E-02
FUCA2	Alpha-L-Fucosidase 2	Hydrolyze the alpha-1,6-linked fucose of glycoproteins	Fucosidosis and Skin Hemangioma	1.3780	1.06E-03
GAA	Alpha Glucosidase	Degrades glycogen to glucose in lysosomes	Glycogen storage disease II, aka Pompe’s disease, Glycogen Storage Disease Due To Acid Maltase Deficiency, Late-Onset	1.0637	2.45E-03
GALNS	N-acetylgalactosamine-6-sulfatase	Hydrolysis of the 6-sulfate groups of the N-acetyl-D-galactosamine 6-sulfate units of chondroitin sulfate	Mucopolysaccharidosis, Type Iva and Mucopolysaccharidosis Iv	1.2107	2.45E-03
GGH	Gamma-Glutamyl Hydrolase	Hydrolyzes folylpoly-gamma-glutamates and antifolylpoly-gamma-glutamates	Tropical Sprue and Pulmonary Neuroendocrine Tumor	1.3750	1.23E-03
GLA	Galactosidase Alpha	Hydrolyses the terminal alpha-galactosyl moieties from glycolipids and glycoproteins	Fabry Disease and Hypertrophic Cardiomyopathy	0.4927	4.43E-02
GUSB	Glucuronidase Beta	A lysosomal hydrolase that degrades glycosaminoglycans, including heparan sulfate, dermatan sulfate, and chondroitin-4,6-sulfate	Mucopolysaccharidosis, Type Vii and Mucopolysaccharidosis, Type Vi	1.4460	1.99E-04
HEXA	Hexosaminidase A	Degradation of GM2 gangliosides in the presence of GM2A	Tay-Sachs Disease	0.6767	3.56E-02
HEXB	Hexosaminidase B	Degrades ganglioside GM2, and other molecules containing terminal N-acetyl hexosamines	Sandhoff Disease and Gm2 Gangliosidosis	1.3787	6.82E-04
IDUA	Alpha-L-Iduronidase	Hydrolyzes the terminal alpha-L-iduronic acid residues of two glycosaminoglycans, dermatan sulfate and heparan sulfate	Hurler Syndrome and Scheie Syndrome	0.6527	3.27E-02
LGMN	Legumain	A cysteine protease that degrades internalized EGFR	Aneruptive Fever and Cerebral Amyloid Angiopathy, Cst3-Related	0.7823	1.68E-02
MAN2B1	Mannosidase Alpha Class 2B Member 1	Hydrolyzes terminal, non-reducing alpha-D-mannose residues in alpha-D-mannosides	Mannosidosis, Alpha B, Lysosomal and Methylmalonic Aciduria Due To Methylmalonyl-Coa Mutase Deficiency	0.4447	8.58E-02
MAN2B2	Mannosidase Alpha Class 2B Member 2	Involved in mannose metabolic process	Mannosidosis, Beta A, Lysosomal and Mannosidosis, Alpha B, Lysosomal	1.0883	1.98E-03
MANBA	Mannosidase Beta	A lysosomal glycosyl hydrolase that degrades N-linked oligosaccharide	Mannosidosis, Beta A, Lysosomal and Angiokeratoma	0.9397	6.16E-03
NAGA	Alpha-N-Acetylgalactosaminidase	A lysosomal enzyme that cleaves alpha-N-acetylgalactosaminyl moieties from glycoconjugates	Kanzaki Disease and Schindler Disease, Type I	1.0667	7.18E-03
NAGLU	N-Acetyl-Alpha-Glucosaminidase	A lysosomal enzyme that degrades heparan sulfate	Charcot-Marie-Tooth Disease, Axonal, Type 2V and Mucopolysaccharidosis, Type Iiib	0.8983	9.05E-03
PLA2G15	Phospholipase A2 Group XV	Hydrolyzes lysophosphatidylcholine to glycerophosphorylcholine and a free fatty acid		1.0920	5.25E-04

These enzymes are crucial for various cellular processes, and their misplacement can lead to aberrant extracellular activity. For instance, cathepsins are involved in protein degradation, and their uncontrolled release may contribute to aberrant extracellular proteolysis. Hexosaminidases, such as HEXA and HEXB, are essential for the breakdown of glycolipids, and their mislocalization may disrupt normal cellular processes and cause Tay-Sachs disease (Barritt et al., 2017; Ramani and Parayil Sankaran, 2023). Additionally, the extracellular presence of lysosomal enzymes could lead to altered interactions with the extracellular matrix and neighboring cells, potentially impacting tissue homeostasis and contributing to pathological conditions (Abbott et al., 2010; Ibata and Yuzaki, 2021; Stoka et al., 2023). This holistic analysis provides novel insights into how GRASP55 and the Golgi structure influence the secretome, shedding light on its role in lysosomal protein trafficking and secretion. Understanding the consequences of the extracellular secretion of these enzymes is crucial for unraveling the broader implications of Golgi defects in lysosomal enzyme trafficking.

### 4.3 The role of GRASP55 in autophagosome-lysosome fusion and unconventional secretion

In addition to the function of the Golgi in lysosome biogenesis, the Golgi stacking protein GRASP55 also plays a role in stress-induced autophagy and unconventional secretion (Zhang and Wang, 2020b). Under conditions of glucose and amino acid starvation, there is a significant increase in the expression levels of the GRASP55 protein (Zhang et al., 2019b), with a subset of the GRASP55 protein localized to autophagosomes (Zhang et al., 2018). This process is regulated by O-GlcNAcylation, a form of glycosylation that serves as a marker for glucose levels. Under growth condition, GRASP55 is O-GlcNAcylated by the O-GlcNAc transferase (OGT). Upon glucose starvation, GRASP55 is de-O-GlcNAcylated and targeted to autophagosomes (Figure 3) (Zhang, 2023).

Interestingly, this autophagosome localization of GRASP55 is accompanied by an enhanced fusion between autophagosomes and lysosomes. This phenomenon was observed through the increased colocalization of LC3 and LAMP2 (Zhang et al., 2018). Conversely, autophagosome-lysosome fusion was notably reduced in cells where GRASP55 was depleted, as evidenced by a significant decrease in LC3 and LAMP2 colocalization (Zhang et al., 2018). In contrast, the expression levels of LC3 and the protein Sequestosome one (p62/SQSTM1), classical selective autophagy receptors, were significantly elevated (Zhang et al., 2018), indicating a reduced autophagic flow.

Mechanistically, GRASP55 interacts with and stimulates the assembly of the Beclin-1-Phosphoinositide 3-kinases (PI3K)-UVRAG complex, which is crucial for the fusion of autophagosomes with lysosomes (Figure 3) (Zhang et al., 2019b). Subsequent studies demonstrated that adding recombinant GRASP55 into cell lysates increased LC3 and LAMP2 complex formation assessed by co-immunoprecipitation (Zhang et al., 2018). Like its role in Golgi stacking, GRASP55 oligomers act as membrane tethers, facilitating fusion by physically connecting autophagosomes and lysosomes via interactions with LC3 (autophagosomes) and LAMP2 (late endosomes/lysosomes) (Figure 3) (Zhang and Wang, 2018b; a).

Initially, GRASPs were found to be associated with the unconventional secretion of acyl-CoA binding protein (AcbA/Acb1) in *Dictyostelium discoideum* and *Saccharomyces cerevisiae* (Kinseth et al., 2007; Manjithaya et al., 2010). Subsequently, cytosolic proteins without ER signal sequences (leaderless proteins), such as the cytokine interleukin one beta (IL-1β) (Chiritoiu et al., 2019), growth factors including fibroblast growth factor 2 (FGF2), as well as some integral membrane proteins (Kinseth et al., 2007; Gee et al., 2011), were all found to be secreted in a Golgi-independent but GRASP-dependent manner (Zhang and Wang, 2020b). In *Drosophila*, the singular GRASP protein was found to be involved in the unconventional trafficking of α-integrin during specific stages of fly development (Schotman et al., 2008). In *Drosophila* adult fat body cells Unpaired 2 (Upd2), analogous to the primary human adipokine leptin, is also secreted through GRASP unconventional secretion pathway (Rajan et al., 2017).

Interestingly, a recent study has shown that GRASP55 is a direct substrate of mTORC1. When mTOR1 is active, it phosphorylates GRASP55 proteins at the Golgi cisternae, where they maintain their cellular localization. Conversely, reduced mTORC1 activity, due to various stresses or inhibitors, results in GRASP55 dephosphorylation, prompting its relocation from the Golgi to autophagosomes and multivesicular bodies (MVBs), leading to the stimulation of unconventional secretion of proteins. This suggests that the mTORC1-GRASP55 signaling axis plays an essential role in controlling the section of extracellular proteome (Nuchel et al., 2021).

Recent studies demonstrated that more cytosolic proteins, including neurodegenerative proteopathic proteins α-synuclein, TDP-43, SOD1, and tau (El-Agnaf et al., 2003; Lee et al., 2005; El-Agnaf et al., 2006; Gomes et al., 2007; Emmanouilidou et al., 2010; Alvarez-Erviti et al., 2011; Grey et al., 2015; Iguchi et al., 2016; Cruz-Garcia et al., 2017; Liu et al., 2017; Ruan and Ikezu, 2019), can be secreted. Studies utilizing mutant huntingtin (mHtt) as a model protein have revealed the potential mechanism for cytoplasmic proteolytic proteins to be secreted through a Golgi-independent, autophagy-dependent, and stress-induced unconventional protein secretion pathway (Ahat et al., 2022a). GRASP55 regulates the secretion and aggregation of mHtt by controlling this pathway (Ahat et al., 2022a). This process involves transporting cargo proteins from the cytosol to autophagosomes and, ultimately, to the extracellular space (Figure 3) (Zhang and Wang, 2020b). Healthy cells can internalize these secreted proteopathic proteins, leading to the spread of disease proteins between cells, resulting in cell death (Sung et al., 2001; Luk et al., 2009; Ruan and Ikezu, 2019; Jo et al., 2020).

The secretome analysis comparing WT and 55KO cells has identified new candidates involved in GRASP55-dependent unconventional secretion (Ahat et al., 2022a). Among the 445 proteins affected significantly by 55KO, 196 (71%) of the 276 proteins lacking ER signal sequences showed decreased secretion in 55KO, underscoring the significance of GRASP55 in unconventional protein secretion. Gene Ontology analysis revealed numerous pathways affected by 55KO, including lysosomal enzymes, extracellular matrix organization, glycosaminoglycan metabolism, and stress response, thereby reinforcing the connection between GRASP55, lysosomal function, and stress response (Ahat et al., 2022a).

Moreover, the secretome analysis has confirmed the secretion of proteins lacking ER signal sequences through a GRASP55-dependent mechanism. Studies of specific candidates such as transgelin 1 (TAGLN), multifunctional protein ADE2 (PAICS), and peroxiredoxin-1 (PRDX1) have demonstrated their secretion dependency on GRASP55 (Ahat et al., 2022a). Furthermore, these studies have underscored the vital roles played by GRASP55 in unconventional protein secretion, providing insights into its effects on cellular processes and emphasizing its connection to lysosomal function and stress response. Despite the identification of numerous substrates in GRASP55-dependent unconventional secretion, a direct interaction between GRASP55 and cargo molecules has not been documented. The mechanisms through which GRASP55 detects cellular stresses, recruits cytosolic proteins, and facilitates their extracellular release remain largely unresolved questions.

### 4.4 Non-classical LSDs, neurodegenerative disorders, and their association with Golgi defects

While classical LSDs are usually associated with inherited mutations in lysosomal enzymes, non-classical LSDs may involve abnormalities in lysosomal function, lysosomal membrane proteins, or other processes related to lysosomal biology. Non-classical LSDs often present with a broad spectrum of symptoms and may not follow the well-defined patterns seen in classical cases. These disorders may result from genetic mutations affecting various aspects of lysosomal physiology, such as lysosomal membrane stability, membrane transporters, or regulatory proteins (Ferreira and Gahl, 2017; Platt et al., 2018). These so called non-classical LSDs represent a diverse group of disorders characterized by the abnormal accumulation of substances within lysosomes, leading to cellular dysfunction (Bajaj et al., 2019; Bonam et al., 2019). The atypical nature of non-classical LSDs poses challenges in their diagnosis and classification, as they may exhibit overlapping features with other metabolic or neurodegenerative disorders (Machtel et al., 2023).

An intriguing connection has been observed between some non-classical LSDs and Golgi defects. These disorders often involve disruptions in cellular trafficking and sorting mechanisms, impacting the proper functioning of the Golgi apparatus (Robenek and Schmitz, 1991; Bajaj et al., 2019). Due to its pivotal role in protein modification, sorting, and transport, any compromise in Golgi function can result in abnormalities in the processing and trafficking of lysosomal enzymes (Figure 3) (Rajkumar and Dumpa, 2023).

One example of non-classical LSDs associated with trafficking defects within the Golgi is sialic acid storage diseases, such as Salla disease and infantile sialic acid storage disease (ISSD) (Ferreira and Gahl, 2017). These disorders are characterized by the accumulation of sialic acid in lysosomes, leading to neurodevelopmental abnormalities. Studies have shown that mutations in the SLC17A5 gene, which encodes sialin, a lysosomal sialic acid transporter, result in the trapping of these proteins in the Golgi, preventing their delivery to lysosomes (Aula et al., 2002). This disruption in SLC17A5 trafficking contributes to the lysosomal storage phenotype observed in these diseases.

Although neurodegenerative diseases are generally not considered classical LSDs, many of them are characterized by protein aggregations and lysosomal dysfunction (Darios and Stevanin, 2020; Root et al., 2021; Udayar et al., 2022). In AD, intracellular NFTs formed by hyperphosphorylated tau are strongly linked to neuronal loss and cognitive decline (Umeda et al., 2014). In Parkinson’s disease (PD) and Lewy body dementia (DLB), α-synuclein forms aggregates known as Lewy bodies (Stefanis, 2012). In ALS and frontotemporal dementia (FTD), TDP-43 protein undergoes abnormal cellular localization and aggregation in the nucleus and cytoplasm (Ross and Poirier, 2004; Johnson et al., 2009; Udan-Johns et al., 2014; Ishii et al., 2017). The aggregation of these proteopathic proteins disrupts normal cellular processes, including protein homeostasis, intracellular signaling, and neuronal function, ultimately leading to neuronal cell death. As discussed above, all these aggregative proteins are released by GRASP55-dependent unconventional secretion.

Neurons maintain high basal autophagy for survival (Feng et al., 2014; Galluzzi et al., 2014; Yin et al., 2016). Autophagy-lysosome defects occur in early AD pathogenesis (Nixon et al., 2005; Levine and Kroemer, 2008) and likely contribute to the formation of amyloid plaques and NFTs (Boland et al., 2008). The Aβ peptide is produced during the autophagic turnover of organelles rich in APP, supplied by autophagy and endocytosis (Nilsson et al., 2013). In CA1 pyramidal hippocampus neurons from subjects with early and late-stage AD, there is a notable increase in autophagosome formation and a progressive impairment in autophagy flux (Nixon, 2007). The combination of increased autophagosome formation and defective clearance of Aβ-generating autophagic vacuoles creates conditions favorable for Aβ accumulation in AD (Nixon, 2007). The sustained activation of autophagy amid declining lysosomal clearance accounts for the unusually robust autophagic pathology implicated in AD pathogenesis (Bordi et al., 2016; Leeman et al., 2018).

AD is frequently associated with type 2 diabetes mellitus (T2DM) and decreased brain O-GlcNAc levels (Franco and Bronson, 2005; Accardi et al., 2012; Rasool et al., 2014; Arnold et al., 2018); Inhibition of O-GlcNAcase (OGA) has been shown to reduce tau aggregation and Aβ production (Yuzwa et al., 2012; Kim et al., 2013; Yuzwa et al., 2014a; Yuzwa et al., 2014b). O-GlcNAcylation is considered an energy-sensing mechanism and part of a protective stress response (Zachara et al., 2004; Zeidan and Hart, 2010; Bond and Hanover, 2015). Furthermore, studies have indicated that de-O-GlcNAcylated GRASP55 facilitates autophagosome maturation (Zhang et al., 2018) and secretion of mHtt (Ahat et al., 2022a), suggesting an important role for GRASP55 and its O-GlcNAcylation in neurodegeneration (Figure 3). The interplay between Golgi fragmentation, APP processing, Aβ production, lysosomal dysfunction, and GRASP55-mediated unconventional tau secretion represents a significant area for future investigation (Zhang and Wang, 2024).

While the connection between Golgi defects and LSDs has not been extensively studied, Golgi defects are more commonly associated with certain neurodegenerative disorders and conditions affecting cellular trafficking rather than classical or non-classical LSDs. The Golgi is abnormally fragmented in AD, PD, and ALS (Aridor and Balch, 1999; Mizuno et al., 2001; Fujita et al., 2002; Fujita and Okamoto, 2005; Joshi et al., 2014; Umeda et al., 2014; Evin, 2015; Joshi et al., 2015; Joshi and Wang, 2015), whereas disturbances in lysosomal function contribute to synaptic and cognitive decline (Hwang et al., 2019). This suggests impaired Golgi apparatus function may lead to lysosomal dysfunction, resulting in neurodegeneration (Figure 3). Understanding the link between lysosomal dysfunction and Golgi defects provides valuable insights into the underlying cellular mechanisms of these disorders. Targeting the Golgi-related pathways may offer potential therapeutic strategies for managing non-classical LSDs or neurodegenerative disease by addressing the aberrant lysosomal enzyme trafficking and sorting associated with Golgi dysfunction. Further research into the molecular basis of these connections holds promise for developing targeted interventions to alleviate the symptoms and progression of non-classical LSDs, neurodegenerative disorders, and other proteopathies.

## 5 Conclusion and perspectives

In conclusion, this review underscores the pivotal role of the Golgi apparatus in orchestrating lysosome biogenesis and maintaining cellular homeostasis through the precise delivery of lysosomal enzymes. Our focused examination of Golgi Reassembly Stacking Proteins (GRASPs) provides key insights into their impact on Golgi apparatus formation and function, elucidating their critical role in maintaining lysosomal homeostasis. The intricate association between Golgi structure, lysosomes, and the onset of LSDs and neurodegenerative disorders emphasizes the importance of understanding Golgi-related pathways. Notably, neurodegenerative disorders, including Alzheimer’s and Huntington’s, serve as illustrative examples, highlighting the profound consequences of Golgi dysfunction on crucial cellular processes such as protein aggregation and lysosomal dysfunction.

Moreover, the identification of aggregative proteins, secreted through GRASP55-dependent unconventional secretion, sheds light on novel pathways and mechanisms underlying these complex disorders. The interplay between Golgi fragmentation, APP processing, Aβ production, lysosomal dysfunction, and GRASP55-mediated unconventional secretion of tau represents a promising avenue for future research.

Furthermore, the exploration of Golgi dysfunction-induced secretion of lysosomal enzymes adds a new layer of complexity to the cellular processes involved in disease pathogenesis. This review aims to serve as a concise yet comprehensive resource, offering insights into Golgi structure, function, and the implications of disease-related defects. By highlighting Golgi defects as an often-underappreciated contributor to lysosomal dysfunction across various diseases, we seek to enhance comprehension of the intricate interplay between these cellular components.

As we continue to unravel the molecular intricacies of Golgi-related pathways, potential therapeutic strategies for managing LSDs and neurodegenerative conditions may emerge, providing new avenues for targeted interventions to mitigate the impact of these complex disorders. Understanding the nexus between lysosomal dysfunction and Golgi defects provides valuable insights into the cellular mechanisms of these disorders. Targeting Golgi-related pathways emerges as a potential therapeutic strategy for managing non-classical LSDs and neurodegenerative conditions. As research progresses, unraveling the molecular basis of these connections holds promise for developing targeted interventions to alleviate symptoms and impede the progression of these intricate diseases.
